# Gustatory function of sensilla chaetica on the labial palps and antennae of three tortricid moths (Lepidoptera: Tortricidae)

**DOI:** 10.1038/s41598-022-21825-w

**Published:** 2022-11-07

**Authors:** Carles Amat, Frédéric Marion-Poll, Miguel A. Navarro-Roldán, César Gemeno

**Affiliations:** 1 University of Lleida-Agrotecnio-CERCA Center, Av. Alcalde Rovira Roure 191, 25198 Lleida, Spain; 2 Université Paris-Saclay, CNRS, IRD, UMR EGCE, 12 rue 128, 91190 Gif-sur-Yvette, France; 3 Université Paris-Saclay, AgroParisTech, 22 place de l’Agronomie, 91120 Palaiseau, France

**Keywords:** Gustatory system, Peripheral nervous system, Neurophysiology, Chemical ecology

## Abstract

In adult Lepidoptera the labial palps are best known for their role in CO_2_ detection, but they can also bear sensilla chaetica which function is unknown. The number and distribution of sensilla chaetica in labial palps was studied using a bright field microscope. To determine if these sensilla have a gustatory function, we performed single sensillum electrophysiology recordings from palp and antennal sensilla of adult moths of *Cydia pomonella* (L.), *Grapholita molesta* (Busck) and *Lobesia botrana* (Denis and Shieffermüller). Each sensillum was stimulated with 3 doses of one of four test stimulus (sucrose, fructose, KCl and NaCl). Overall, responses (spikes/s^−1^) increased with dose, and were higher in the palps than in the antennae, and higher to sugars than to salts. With sugars the response increased with concentration in the palp but not in the antenna. With salts there was a drop in response at the intermediate concentration. The number and position of sensilla chaetica on labial palps was variable among individuals. Sensilla were located in the most exposed areas of the palp. Differences in sensilla distribution were detected between species. Such differences among species and between palps and antenna suggest that taste sensilla on the palps have an unforeseen role in adaptation.

## Introduction

Reception of gustatory stimuli (also referred to as gustation, taste or contact chemoreception) plays a vital role in many aspects of insect fitness, such as in food selection and oviposition choices^1,2^. Tastants are detected by gustatory receptor neurons (GRNs), which are typically present in groups of 2 to 4 inside gustatory sensilla^2^. Gustatory sensilla have been found in almost any part of the adult body, especially those in direct contact with taste stimuli, such as the tarsi, mouthparts, food channel, ovipositor and antennae^2^. There are different morphological types of gustatory sensilla^2,3^. Sensilla chaetica are long, hair-like structures that can be differentiated from similar sensilla (e.g. sensilla trichoidea) by having thicker walls^3,4^. The presence of a terminal pore in sensilla chaetica is usually associated with gustatory function and a flexible basal socked with tactile function^3^. Sensilla chaetica typically have a contact chemoreception function, with two to four GRNs and one mechanosensory neuron which terminates in a tubular body at the base of the sensilla^4,5^.

Gustation in the mouthparts of adult insects is generally found in the galeae, in the maxillary palps of the maxilla, and in the labial palps of the labium. The labial palps of insects with standard chewing mouthparts (e.g., Coleoptera, Orthopteroids) are usually small and bear gustatory sensilla^6–8^. In insects with mouthpart modified for sucking and licking (e.g., Diptera, Hemiptera), the labium and associated labial palps retain their taste function^9–12^. In Lepidoptera, the galeae have joined in a long and coiled proboscis, and in most lineages the rest of the mouthparts structures are largely reduced, except for the labial palps, which are relatively large and cover most of the front part of the insect head at each side of the proboscis^13^. The labial palps of Lepidoptera present a cavity at the tip of the apical segment, the labial-palp pit organ (LPO), which contains sensilla that respond to changes in CO_2_ concentration^14^. A ventral glomerulus in the antennal lobe receives the input of the CO_2_ sensory cells. CO_2_ detection may help Lepidoptera in host-plant selection^14^. In some moth species, the labial palps bear chaetica-like sensilla^15,16^, which often have a gustatory function in other insect appendages^3–5^. The presence of putative gustatory receptors of the sugar clade in the labial palps of *Helicoverpa armigera* (Hübner)^17^ further indicates the presence of gustation in the labial palps, but as far as we know, there is no physiological evidence that the labial palps of Lepidoptera have a gustatory function. The antennae of Lepidoptera are well known for their olfactory function performed by numerous sensilla trichoidea, but they also bear some gustatory sensilla chaetica in each flagellum^2^. In noctuid moths the gustatory function of antennal sensilla chaetica has been demonstrated^18–20^. The gustatory function of sensilla chaetica on the antennae of tortricid moths^21–23^ remains to be shown.

The objective of this study is to determine if the sensilla present of the surface of the labial palp of moths respond to gustatory stimuli. To this end we performed electrophysiological recordings from adults of three tortricid moths, *Cydia pomonella* (L.)*, Lobesia botrana* (Denis and Shiffermüller) and *Grapholita molesta* (Busck), which are key pest species of fruit trees and vines worldwide^24,25^**.** Two sugars (fructose and sucrose) and two salts (KCl and NaCl) were tested at 3 concentrations. In addition, we tested the response of sensilla chaetica in the antennae. In order to determine the biological relevance of sugars in these species we also studied their effect on the adult longevity.

## Results

### Labial palp sensilla

**Figure 1:**
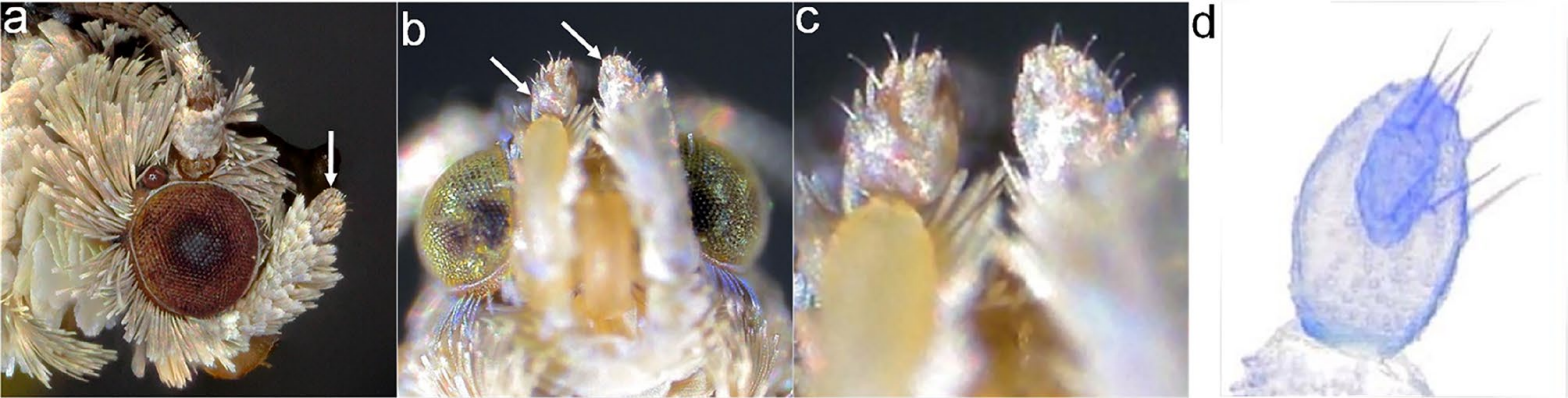
Labial palps of a female *G. molesta*. (**a**) Lateral view, (**b**) ventral view, scales have been partially removed from the right palp, (**c**) close up of "b" showing sensilla chaetica clearly protruding among the scales from the third segment, (**d**) third segment digested with KOH and stained with gencian violet to show the labial pit organ (elongated dark sac inside the palp) and the sensilla chaetica on the surface. White arrows in panels “(**a**)” and “(**b**)” indicate the apical segment of labial palp.

The labial palps were composed of three segments densely covered with scales, with sensilla protruding from the scales in the apical segment (Fig. 1). These sensilla have a typical chaetica morphology, they are hair-like structures relatively straighter and more rigid than sensilla trichoidea, and with a flexible socket at the base^3–5^. The second segment was the longest (*G. molesta* 356.5 µm, *L. botrana* 464 µm and *C. pomonella* 636 µm on average), it was curved upwards and projected forward on each side of the head (Fig. 1). The apical segment was oval, between 120 and 237 µm long and 60 to 143 µm wide depending on the species and sex (Table 1). The apical segment was longest in *C. pomonella*, followed by *L. botrana* and *G. molesta* and widest in *C. pomonella* than in the other two species. It was wider and longer in females than in males (Supplementary Table S1). The number of sensilla in the apical segment of the labial palp ranged between 5 and 15 (Table 1), and it was positively correlated with the area of the segment (Person correlation coefficient = 0.73, t_58_ = 8.15, *p* < 0.001). In general females had 1.3 more sensilla than males and the number of sensilla was 1.3 times higher in *C. pomonella* than in *G. molesta* (Supplementary Table S2).

**Table 1:** Dimensions and number of sensilla chaetica on the apical segment of the labial palp.

Species	Sex	Size (*µ*m, Mean ± SEM)		Number		
		Length	Width	Mean ± SEM	Min	Max
*L. botrana*	Female	237.00 ± 6.01	106.75 ± 1.79	10.00 ± 0.52	7	12
	Male	184.25 ± 2.56	65.00 ± 2.17	7.30 ± 0.37	5	9
*G. molesta*	Female	148.25 ± 4.79	94.00 ± 4.00	7.80 ± 0.61	5	10
	Male	120.50 ± 1.93	79.25 ± 1.35	7.40 ± 0.54	5	9
*C. pomonella*	Female	278.25 ± 7.59	142.25 ± 3.86	12.30 ± 0.54	9	15
	Male	202.00 ± 5.55	116.75 ± 2.14	8.70 ± 0.54	6	12

The position of sensilla chaetica in the apical segment of the labial palp was highly variable between individuals and no consistent location could be determined for any sensillum. However, some general patterns could be observed. Sensilla tended to be located on the most exposed areas (of the resting palp). In addition, many sensilla occurred around the LPO opening (39% in *L. botrana,* 30% in *C. pomonella* and 23% in *G. molesta*), (Fig. 1, Supplementary Fig. S1). In all species, more than 80% of sensilla were located on the distal half of the segment and more than half of the sensilla (62% in *L. botrana*, 66% in *G. molesta* and 75% in *C. pomonella*) were lateral*.* The most substantial difference between species was found between dorsal and ventral regions. In *G. molesta* 90% of the sensilla were ventral, while in the other two species the number of sensilla in the dorsal and ventral regions was similar (Supplementary Fig. S1, Supplementary Table S3). The results of the ANOVA of the distribution of the sensilla among the areas defined by anatomical symmetry axes showed differences among species (Deviance = 106.28, df = 14, *p* [Chi] < 0.001), but not between sexes (Deviance = 11.53, df = 7, *p* [Chi] = 0.117) and no interaction between species and sexes was detected (Deviance = 10.23, df = 14, *p* [Chi] = 0.745). Within each species, the number of sensilla varied between areas. The ventro-lateral-distal area of the palp had the highest number of sensilla in all species, however substantial differences could be observed between species in sensilla distribution among areas (Tukey: *p* < 0.05, Supplementary Fig. S1, Supplementary Table S3).

### Effect of diet on survival

Diet had a significant impact on adult survival in all three species (Log-rank test: *p* < 0.0001), the longest survival time was detected in *C. pomonella* with access to sugared water (35 days) and the lowest in *G. molesta* without water (1 day). In the absence of water, median longevity decreased by 75% in *G. molesta,* 50% in *L. botrana* and 40% in *C. pomonella*. Addition of sugar to the diet increased median longevity in *G. molesta* and *C. pomonella* by 6 and 4.5 days respectively (Log-rank test: *p* < 0.0001, Supplementary Fig. S2).

### Electrophysiology

**Figure 2:**
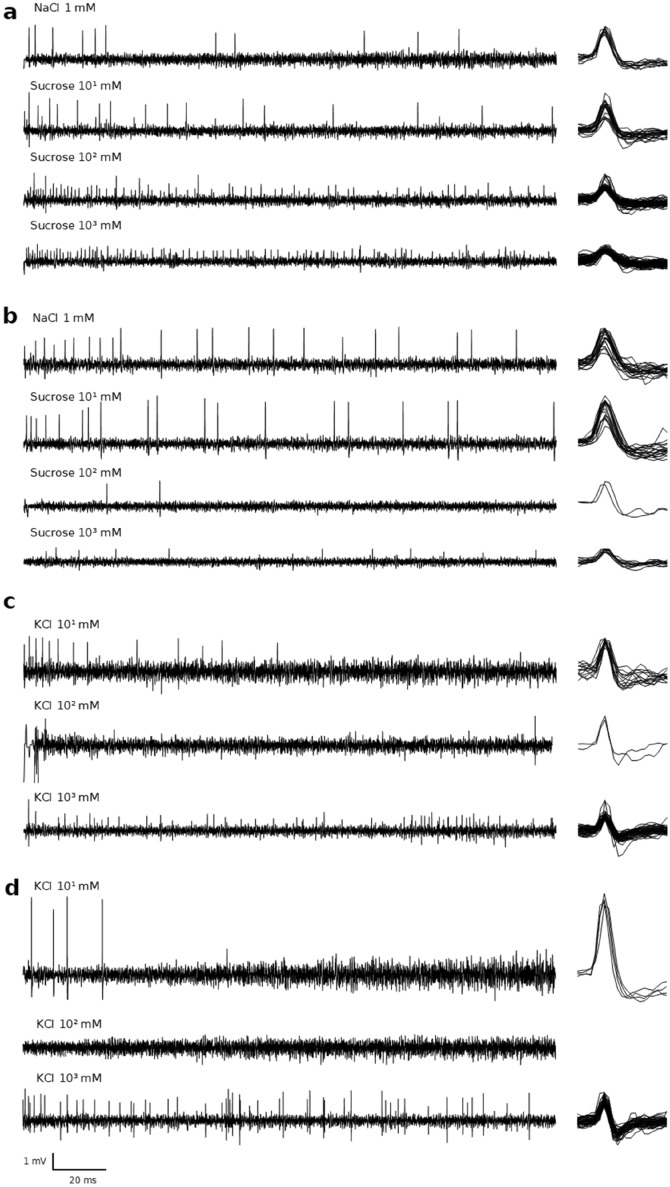
Representative single-sensillum recording traces (left) of sensilla chaetica and superimposed spikes (right) detected in the recording (6 ms section). Each group (**a**–**d**) shows the response of the same sensillum to increasing concentrations of test stimulus. (**a**) Labial palp of *C. pomonella* female, (**b**) antenna of *C. pomonella* male, (**c**) labial palp of *C. pomonella* female, and (**d**) antenna of *L. botrana* male. NaCl 1mM=electrolite.

**Figure 3:**
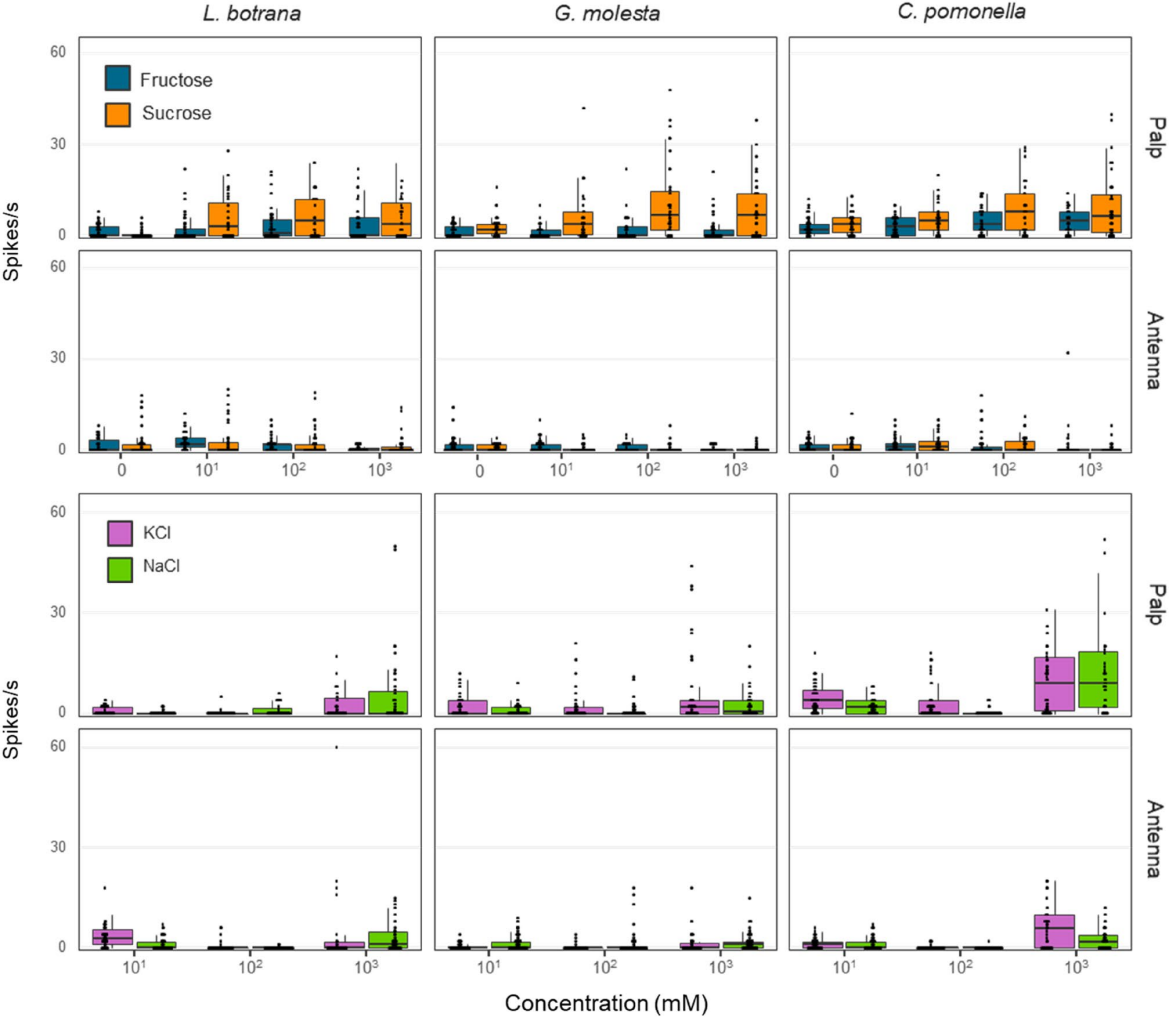
Response of gustatory receptor neurons of sensilla chaetica located on the apical segment of the labial palps and antenna of three tortricid moth species upon a 2 s stimulation with three doses of NaCl, KCl, fructose or sucrose. N = 16–34 sensilla of 4–6 individuals of each sex. Males and females have been combined in this plot. Box plots show median (horizontal line), first and third quartile (box), 1.5*inter-quartile range (error bars), and data points (•).

For each combination of factors (stimulus, species, sex and appendage) between 16 and 34 sensilla from between 4 and 6 individuals were sampled, with a total of 3690 recordings from 1057 sensilla sampled from antenna and palp together (Supplementary Table S4). The highest number of spikes/s (68) was observed on the labial palp of a female *C. pomonella* in response to KCl*,* but in general the number of spikes was relatively low (2.84 on average) (Fig. 3). The average spike amplitude was relatively variable between recordings of the same sensillum and between sensilla (Fig. 2). Thus, although several spike amplitude classes could be detected in some recordings, we did not sort spikes. Instead, for the purpose of this study, we analyzed the sum of all the spikes in a given recording, irrespective of whether they belong to one or more neuron types. Different number of spikes between electrolyte and sugars indicate that the electrolyte alone did not stimulate sensilla more than the test stimuli (Dunnett test: *p* < 0.01, Supplementary Table S5).

The model that best explained responses to stimuli contain all main factors with a 4th-order interaction (Likelihood-ratio test: *p* < 0.01, Supplementary Table S6). According to the ANOVA (Table 2), appendage (i.e., antenna or palp) explained 23.95% of the deviance, the interaction between stimulus and concentration explained 13.07% of the deviance, concentration 9.98%, stimulus 7.77%, and species 6.8%. The remaining 25 factors of the model together explained 33.56% of the deviance, each contributing 4.79% or less to the total deviance. On average, response was 2.76 times higher in palpi than in antennae, and the number of spikes increased with stimulus concentration (Tukey: *p* < 0.05, Table 3). There were 1.2 times more spikes/s to sugars (fructose and sucrose combined) than to salts (NaCl and KCl combined), and sucrose was the strongest of the four stimuli tested (4.30 ± 0.26 spikes/s) (Tukey: *p* < 0.05, Table 3, Fig. 3). *C. pomonella* produced 1.8 times more spikes/s than *G. molesta* (Tukey: *p* < 0.05, Table 3, Fig. 3). Sex had a significant, but very low, impact on the number of spikes (0.76 more in females than in males, Tukey: *p* < 0.05, Table 3), and so the two sexes were combined for graphic display (Fig. 3).

**Table 2:** Summary of the GLM model for the effect of species (*C. pomonella*, *G. molesta* and *L. botrana*), sex, stimulus (fructose, sucrose, NaCl and KCl), dose (10^1, 10^2, 10^3 mM), and appendage (antenna or labial palp) on the number of action potentials of sensilla chaetica. Terms have been ordered by their contribution to the model deviance (% Dev.).

Model term	Df	Deviance	Resid. Df	Resid. Dev	F	*p*(> F)	% Dev	Cum. Dev
NULL			3170	23,956				
Appendage	1	2125.63	3166	21,073	334.77	< 0.001	23.95	
Stimulus*dose	6	1160.41	3130	16,776	30.46	< 0.001	13.07	37.02
Dose	2	885.62	3161	19,497	69.74	< 0.001	9.98	47.00
Stimulus	3	690.08	3163	20,383	36.23	< 0.001	7.77	54.77
Species	2	612.75	3168	23,343	48.25	< 0.001	6.90	61.67
Appendage*stimulus	3	422.64	3138	18,122	22.19	< 0.001	4.76	66.44
Species*appendage*stimulus	6	378.64	3112	15,984	9.94	< 0.001	4.27	70.70
Species*sex*stimulus	6	355.39	3122	16,420	9.33	< 0.001	4.00	74.71
Species*stimulus	6	255.47	3151	18,889	6.71	< 0.001	2.88	77.58
Species*appendage	2	240.03	3157	19,145	18.90	< 0.001	2.70	80.29
Species*dose	4	192.58	3147	18,697	7.58	< 0.001	2.17	82.46
Appendage*dose	2	185.58	3136	17,936	14.61	< 0.001	2.09	84.55
Species*sex*appendage*stimulus	6	168.81	3073	15,370	4.43	< 0.001	1.90	86.45
Sex	1	144.24	3167	23,199	22.72	< 0.001	1.62	88.07
Species*stimulus*dose	12	140.23	3096	15,769	1.84	0.037	1.58	89.65
Species*sex	2	112.59	3159	19,385	8.87	< 0.001	1.27	90.92
Species*sex*stimulus*dose	12	111.86	3057	15,214	1.47	0.129	1.26	92.18
Appendage*stimulus*dose	6	103.15	3079	15,538	2.71	0.013	1.16	93.34
Sex*stimulus*dose	6	90.26	3085	15,642	2.37	0.028	1.02	94.36
Sex*stimulus	3	89.22	3143	18,544	4.68	0.003	1.01	95.37
Species*appendage*stimulus*dose	12	87.17	3045	15,127	1.14	0.319	0.98	96.35
Species*appendage*dose	4	74.88	3108	15,909	2.95	0.019	0.84	97.19
Sex*appendage	1	63.07	3146	18,634	9.93	0.002	0.71	97.90
Species*sex*dose	4	56.60	3118	16,363	2.23	0.064	0.64	98.54
Sex*appendage*stimulus*dose	6	47.63	3039	15,079	1.25	0.277	0.54	99.08
Species*sex*appendage*dose	4	43.91	3069	15,326	1.73	0.141	0.49	99.57
Sex*appendage*stimulus	3	34.78	3093	15,734	1.83	0.140	0.39	99.96
Sex*appendage*dose	2	2.47	3091	15,732	0.19	0.823	0.03	99.99
Species*sex*appendage	2	0.72	3128	16,775	0.06	0.945	0.01	100.00
Sex*dose	2	0.05	3141	18,544	0.00	0.996	0.00	100.00

**Table 3:** Pairwise comparison of the number of spikes produced by sensilla chaetica present on the antennae and labial palps of the three moth species. Observed data and estimated values from the GLM model are presented. Letters in the right column indicate significant differences among the levels of each factor (Tukey ´s test, *p* < 0.05 after GLM).

	Observed	Estimated	
	Mean ± SEM	Mean ± SEM	
**Appendage**			
Palp	4.43 ± 0.19	2.93 ± 0.15	a
Antenna	1.61 ± 0.09	0.80 ± 0.13	b
**Concentration (mM)**			
10^3	4.33 ± 0.25	2.69 ± 0.18	a
10^1	2.37 ± 0.12	1.69 ± 0.12	b
10^2	2.30 ± 0.16	0.79 ± 0.18	c
**Stimulus**			
Sucrose	4.30 ± 0.26	2.68 ± 0.21	a
Fructose	2.33 ± 0.14	1.65 ± 0.14	b
KCl	3.13 ± 0.24	1.24 ± 0.30	b
NaCl	2.32 ± 0.20	1.00 ± 0.20	b
**Stimulus group**			
Sugar	3.28 ± 0.15	2.10 ± 0.12	a
Salt	2.72 ± 0.16	1.11 ± 0.18	b
**Species**			
*C. pomonella*	4.09 ± 0.22	2.11 ± 0.25	a
*L. botrana*	2.66 ± 0.16	1.51 ± 0.18	ab
*G. molesta*	2.28 ± 0.17	1.12 ± 0.13	b
**Sex**			
Female	3.38 ± 0.15	1.87 ± 0.16	a
Male	2.63 ± 0.15	1.25 ± 0.17	b

The effect of dose was different for salts and sugars. In salts the highest response occurred at 1 M, then at 10 mM and the lowest at 100 mM (Tukey: *p* < 0.05, Supplementary Table S6, Fig. 3). With sugars, no differences were found among concentrations when pooling antennal and palp responses (Tukey: *p* < 0.05, supplementary Table S6). However, in antenna the response to sugars decreased as the dose increased, whereas in the palp responses increased with concentration (Tukey: *p* < 0.05, Supplementary Table S6, Figs. 2 and 3).

From highest to lowest, the mean responses to sugars and salts were as follows: sugars in palps (5.33 ± 0.27 spikes/s), salts in palps (3.60 ± 0.27 spikes/s), and sugars and salts in antennae (1.40 ± 0.11 and 1.81 ± 0.14 respectively) (Tukey: *p* < 0.05, Supplementary Table S6, Fig. 3). Responses to sugars in *C. pomonella* and *L. botrana* were in average 1.9 times higher than in *G. molesta,* but no differences were detected between species with salts. Females produced more spikes than males in *L. botrana* and *G. molesta* (Tukey: *p* < 0.05, Supplementary Table S6).

## Discussion

We present evidence that the labial palps of moths have a gustatory function. Furthermore, we found significant differences between appendages, stimuli, species and sexes. As far as we know, this may be the first direct evidence that the labial palps of moths, or perhaps any Lepidoptera, have a gustatory function, while it is a common feature in other insect orders. The presence of a flexible socket at the base of the sensilla chaetica suggests mechanosensory function^3,4^, however observations should be conducted to confirm this role. We do not know the extent of gustation in the labial palps of Lepidoptera because reports of uniporous sensilla chaetica (i.e., with a putative gustatory function) on the external part of labial palps are scarce. Chaetica-like sensilla with a terminal pore have been reported in a species of the Cossidae family^16^. Sensilla chaetica are described in other Cossidae and in members of the Tortricidae and Pyralidae families, but it is not indicated if they bear a pore at the tip, and thus they may not be gustative^15,26,27^. In a Neopseustidae specie, aporous sensilla chaetica have been described^28^**.** Several studies on members of the Gelechiidae, Pyralidae, Sphingidae, Noctuidae, Plutellidae, Erebidae and Nymphalidae families fail to report sensilla chaetica or any other type of sensilla with a putative gustatory function in the labial palps^29–38^.

Obtaining electrophysiological recordings from sensilla chaetica of the labial palps and antenna was challenging because we experienced a persistent difficulty to make stable electric contacts with these sensilla, whereas it was relatively easy on sensilla styloconica of the proboscis (data not shown). Conditions improved when the humidity of the rearing chamber was increased, but consistent contacts were only obtained when the sensilla tip was wiped repeatedly with an empty glass capillary before making contact. Some authors have encountered similar problems in other insects and suggest that debris accumulates on the pore at the tip of the sensilla. Städler et al.^39^ report that low relative humidity reduced the quality of the recordings of *Rhagoletis cerasi* L. tarsi, and that flies that were allowed to walk on the plants were easier to record (implying that the sensilla were "unplugged" by the contact with the substrate). Canney and Gardner^40^ report that cleanness of the pore of sensilla chaetica improved the quality of electrophysiological recordings in *Ostrinia nubilalis* (Hübner).

Sugars and salts play a role on the fitness and behavior of the three tortricid moths. Our longevity test confirms that adults of the three species do not live long without water and that sugars prolong longevity^41–43^. When given access to flowers under laboratory conditions the longevity of *C. pomonella* increased with respect to a no-food control, but a dilution of sugar in water was even better than the flowers^44^. Peach extrafloral nectar, a natural solution rich in sugars^45^, enhanced *G. molesta* adult longevity^42^. Despite the importance of sugar on adult fitness, only *L. botrana* has been observed feeding in the wild. It visits the flowers of tansy (*Tanacetum vulgare* L.) (a non-host plant located around the vineyards) at dusk to feed on the pollen and nectar^46^. Sugars stimulate oviposition in *C. pomonella* and *L. botrana*^47,48^, and gustatory sensilla in the ovipositor of *L. botrana* detect fructose and sucrose^48^. KCl is also perceived by sensilla in the ovipositor of *L. botrana,* and it affects oviposition^48^. Sugars and salts could impact larval fitness too^49–51^. Therefore, the presence of taste receptors on antennae and palps could contribute to the detection of salts and sugars, but they may also be specialized on the detection of other stimuli not tested yet.

Gustatory sensilla of insects typically have 4 different GRNs, generally associated to different taste modalities^2,3^. GRNs in the antennae of adult Lepidoptera respond to sugars, water, salts, amino acids and bitter compounds^18–20^. The number of spikes that we observed in tortricids was relatively lower than what has been found in antennal sensilla of noctuid moths^18–20^. We also observed that the response to sugars in antennal sensilla decreased as concentration increased, contrary to what has been observed in *Chloridera* (formely *Heliothis*) *virescens* (Fabricus) and *Spodoptera littoralis* (Boisduval)^19,20^. Many noctuids are flower visitors, but this behavior is rare in tortricids^52^, and so different sugar requirements between members of the two families may explain differences in sugar sensitivity. Water and salt intake regulation is crucial for animals to maintain osmotic homeostasis^1,53^, and in many insects some cells respond to low salt concentrations (water GRN) and some to high salt concentrations (salt GRN), which allows for fine detection of salts^19,20,53^**.** In our tests, the relatively high number sensilla responding to the electrolyte control, and the unusual shape of the dose–response curve to salts (with a drop at the intermediate concentration) suggests that palp and antennal sensilla chaetica of tortricids have a water and a salt GRN, which may help in salt detection.

There are few reports on the putative behaviors where Lepidoptera may use the gustatory sensilla detected on the labial palps. Grant^54^ observed the response of the tortricid moth *Choristoneura fumiferana* (Clemens) to a rubber septum loaded with female sex pheromone. The tips of the labial palps contacted with the septum ("the palps themselves pulled away from the head in lever-like fashion") right before attempting copulation (Fig. 1 in^54^). Also during courtship, *G. molesta* males and females perform a dance where there is mutual contact with the antennae and probably the palps too^55^. Thus, gustatory receptors on palp and antennae may be used to detect conspecific signals, such as cuticular hydrocarbons, which often present sexual dimorphism in insects^56,57^. Gustatory function of the labial palps may also be related to oviposition choice behavior because in the moth *Cactoblastis cactorum* (Berg) females actively touch the plant with the labial palps while searching for oviposition sites^58^.

The discovery of gustation in the labial palps of moths may shed new light on the innervation of the labial palp nerves in the moth brain. Unilateral palp backfills typically reveal bilateral projections ascending to the antennal lobe where they stain a basal glomerulus, equivalent in both males and females, the "labial pit organ glomerulus" (LPOG)^33,59,60^. This glomerulus receives the input from the CO_2_ sensory neurons housed in the labial pit organ. However, palp backfills show additional arborizations in the suboesophageal ganglion (SOG)^33,59^. To clarify this point, Pramod et al.^60^ performed selective mass staining from both, the inside of the labial pit organ and the outer surface of the distal labial palp segment of *H. armigera* and compared their arborizations. LPO sensory neurons projected exclusively to the LPOG, whereas the non-LPO sensory neurons targeted the gnathal ganglion and the ventral nerve cord. It is very likely that the axons that do not innervate the LPOG correspond to mechano/gustatory neurons located on gustatory sensilla because the mechano/gustatory input from other cephalic appendages ends in the SOG^2,3^. Interestingly, an SEM picture of the labial palp of *H. armigera* does not show sensilla chaetica^33^. Sensilla chaetica are rather conspicuous and resilient^61^ so it is unlikely that they are accidentally knocked off when scales were removed to observe the palps. If they did not fall during scale removal, then other type of sensilla may contain the palp neurons that innervate the SOG. Thus, further studies are required to determine which other Lepidoptera species have gustatory function in the labial palps.

## Material and methods

### Insects

Larvae were reared on a semi-artificial diet modified from that of Ivaldi-Sender^62^ at 25 °C under a 16:8 light:dark photoperiod. Pupae were sexed and kept in separate environmental chambers with unrestricted access to a 10% sucrose in water dilution. Before electrophysiological recordings, adults were anaesthetized with CO_2_ to restrain them inside a modified pipette tip, with their head, antennae and palps fixed with melted dental wax (ref: R3712-00, Leone s.p.a., Firenze, Italy). All insects tested were between 1 and 3 days old.

### Labial palp sensilla

To investigate the location of sensilla chaetica in the labial palps, 10 palpi from different individuals were observed for each species and sex in a bright field microscope. We removed the scales, cleared the cuticle and stained the preparations according to George and Nagy^63^. Detached heads were boiled in 10% KOH until the scales fell off and then washed in distilled water and cleared in 2.5% bleach until they became transparent. After a second wash in distilled water, they were immersed in 0.5% crystal violet until the sensilla were clearly visible. Samples were placed on a drop of glycerol on a microscope slide under a cover slip.

The maximum length and width of the apical segment of the labial palp was measured with an eyepiece micrometer. To assert sensilla position, the apical segment was divided in 8 areas (Supplementary Fig. S1) resulting from the intersection of the 3 axes of symmetry (sagittal, transversal and frontal), and the number of sensilla in each area was recorded.

### Electrophysiology

Gustatory stimuli [NaCl (CAS: 7647-14-5, ref: S7653-1 KG), KCl (CAS:7647-14-5, ref: S7653-1 KG), sucrose (CAS: 57-50-1, ref: S9378-1 KG) and fructose (CAS: 50-48-7, ref: F0127-1 KG), Sigma-Aldrich, Madrid, Spain] were diluted in deionized water. Sucrose and fructose dilutions contained 1 mM NaCl as electrolyte. Three concentrations of each stimulus (10, 100, 1000 mM) were prepared in 1 ml aliquots and kept at − 20 °C. During the experiments, one aliquot of each concentration was defrosted and kept at 4 °C for up to one week.

For each individual, two to six sensilla chaetica from either the antenna or the apical segment of the labial palp were tested. The antenna was sampled up to the apical segment. On the antenna, sensilla chaetica were differentiated from sensilla trichoidea by their morphology (straighter and thicker), their orientation (perpendicular to the surface) and their optical characteristics (brighter under the stereo microscope). On the outside of the labial palp only sensilla chaetica were present. Only one stimulus was used for a given individual, starting from lowest to highest concentration. The contact with each sensillum was limited to 2 s, allowing ≥3 min between stimulations to avoid adaptation. Sugars stimuli were preceded by stimulation with the electrolyte (1 mM NaCl) as a control.

Electrophysiological recordings from sensilla chaetica were obtained using the tip recording technique^64^. Glass capillary recording electrodes (Hirschmann Laborgeräte GmbH & Co, Germany) were pulled to obtain a 3.5 μm wide tip (PP-830, Narishige, Japan), which was filled with the test solution and connected to a 0.5 mm-wide platinum wire, fitted to a preamplifier probe (Taste Probe, Syntech, Germany). The recording electrode was placed over the sensillum tip using a micromanipulator (NMN-25, Narishige, Japan) under a stereomicroscope (Leica M125, objective 2x, oculars 25x, zoom range 0.8–10, Leica microsystems, Spain). The reference electrode was a sharpened tungsten wire (0.125-mm diameter, 99.98% purity, Advent Research Materials Ltd, UK), inserted into the eye with the help of a micromanipulator (UM-3C, Narishige, Japan). The recording electrode was filled with the stimulus solution just before stimulation, it was discarded after 2 minutes and replaced by a new one if needed.

The signal from the recording electrode was pre-amplified (10x) and filtered (10 Hz high-pass) using a Taste Probe amplifier (^65^; Syntech, Germany) and further amplified (50x) and filtered (3000 Hz low-pass filter) (AC/DC differential amplifier, A-M systems Inc., WA, USA). The signal was digitalized and analyzed (Micro 1041-3 and Spike2, respectively, Cambridge Electronic Design Limited, UK), and spike detection was performed using dbWave software^66^.

### Effect of water and sucrose on adult longevity

We determined the effect of water and sugar on the survival of 20–24 adults in order to assess the importance of these stimuli on adult fitness. To this end, groups of 4 to 9 newly emerged adults of both sexes picked randomly from the colony were placed in ventilated 100 mL plastic bottles after CO_2_ anesthesia. Four bottles were prepared for each treatment: control (dry cotton swab), water: (a cotton swab soaked in distilled water) and sugar (cotton swab soaked in 10% domestic sucrose diluted in distilled water). Cotton was replaced regularly so that it was never dry. Bottles were inspected daily (with sporadic 2 to 3 day gaps for the longest-lived species, *C. pomonella*) until all individuals were dead, dead individuals were scored and removed.

### Data analysis

Statistical analyses were run in R 4.1.2 software^67^. Generalized lineal models (GLM) were used for all the analyses with specific error families and link functions where needed. Pairwise comparisons used the Tukeys´s test of the package “emmeans”, unless specified otherwise. To analyze length and width of the apical segment of the labial palp a GLM with Gaussian error distribution was used. The total number of sensilla in the apical segment of the labial palps and the distribution of sensilla, within each species, among the delimited areas (Supplementary Fig. S1) was analyzed with GLM with Poisson error distribution and a logit link. For spike counts analysis only the response to stimuli, and not to the 1 mM NaCl electrolyte, was analyzed. A *quasi*-Poisson error distribution was used because overdispersion was detected. A different model was run to compare the number of spikes between each sugar concentration and the electrolyte control using Dunnett test.

Model selection started from the simplest model containing no main effects, then main factors and interactions were added sequentially. For comparison between models, the likelihood ratio test (LRT) and the Akaike information criterion (AIC) were used, and models with lower AIC values and significantly different LRT were selected. The model with the best fit was used to conduct pairwise comparison between relevant groups of significant factors using estimated marginal means.

To check the relationship between number of sensilla and labial palp size, a Pearson correlation test was conducted between total number of sensilla and the surface of the labial palp. The area of the 3^rd^ segment of the labial palp was estimated as the area of a cylinder with diameter equal to the maximum width of the segment and length equal to the length of the segment. The comparison of sensilla distribution among the areas of a labial palp was conducted with a “vector GLM” using the package “VGAM”, an ANOVA was used to calculate the significance level of species and sexes.

The effect of diet on survival curves was calculated using the Kaplan–Meier method^68^, and a log-rank test was used to detect overall differences among curves. Log-rank test and Benjamini–Hochberg correction was used for multiple pairwise comparisons among curves.

## Supplementary Information

Supplementary Information.

## Data Availability

Raw data and R-script for statistical analysis are available online at https://doi.org/10.34810/data222.
