# Supplementary material for: Gustatory function of sensilla chaetica on the labial palps and antennae of three tortricid moths (Lepidoptera: Tortricidae)
Source: Sci Rep. 2022 Nov 7;12:18882. doi: 10.1038/s41598-022-21825-w (PMC9640605; doi:10.1038/s41598-022-21825-w)
Supplement: Supplementary file 1 — Supplementary Information. [file 41598_2022_21825_MOESM1_ESM.pdf]

Gustatory function of sensilla chaetica on the labial palps and antennae of three tortricid moths

Carles Amat, Frédéric Marion-Poll, Miguel A. Navarro-Roldán, César Gemeno

Supplementary material

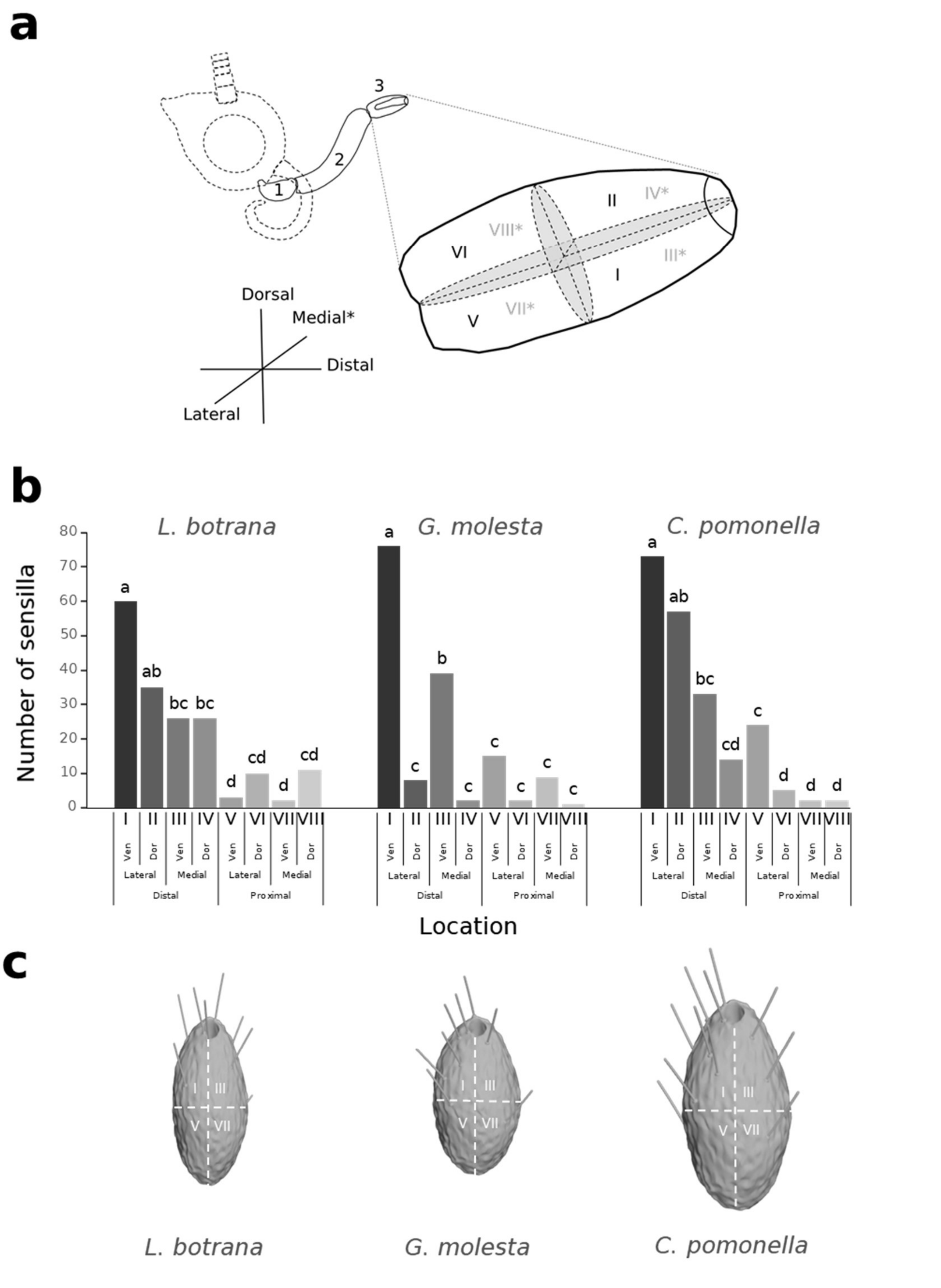

Figure S1. Number and location of sensilla chaetica in the apical segment of labial palps. A) Scheme showing the position of labial palp in the moth head and the areas used to count the sensilla based on the symmetry axes. B) Number of sensilla found in each of the areas. Different letters indicate significant differences among groups (Tukey´s test, P<0.05 after GLM). Males and females have been combined in this plot. C) Schematic diagram showing the sensilla distribution on a ventral view of the labial palps.

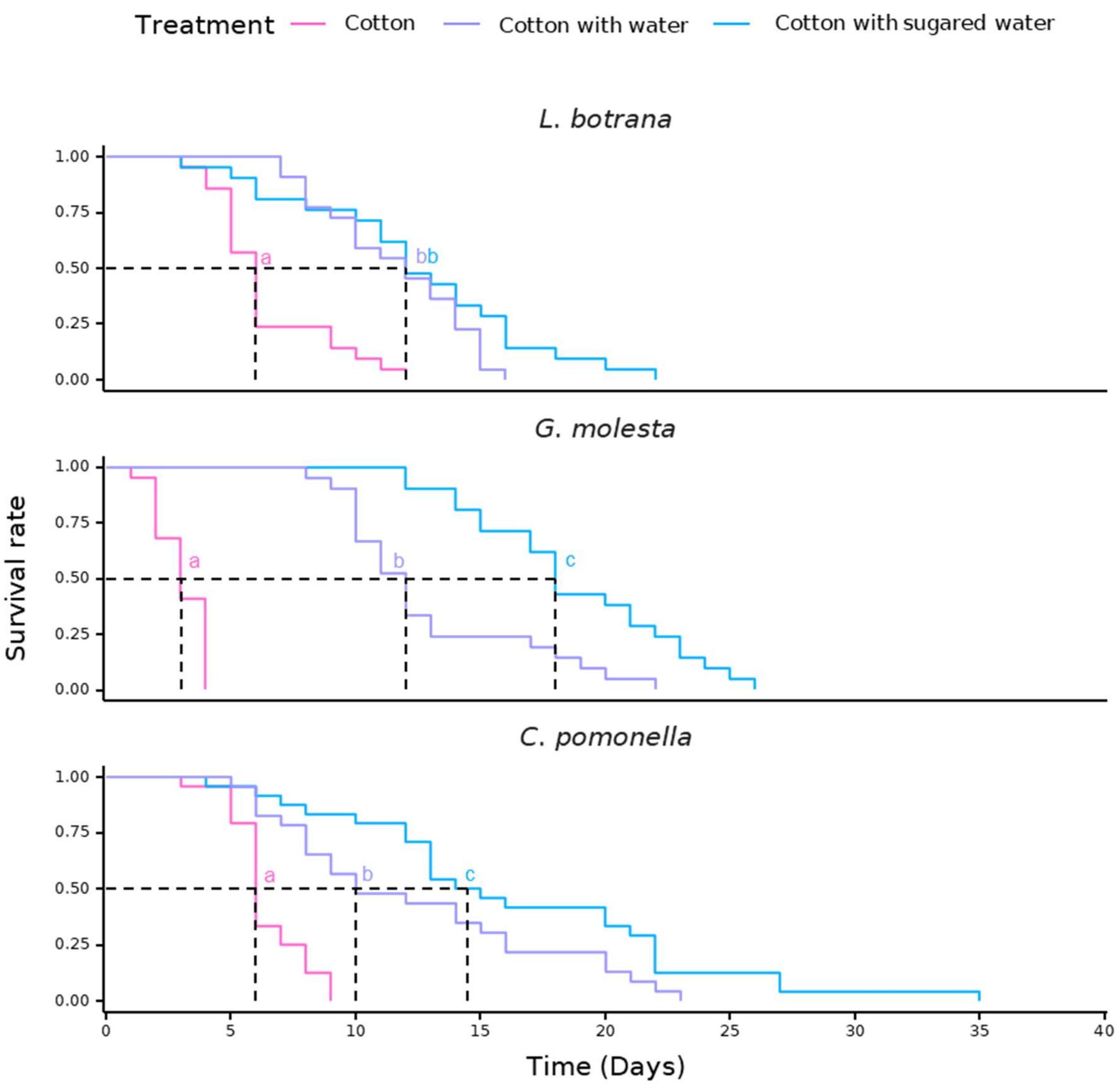

Figure S2. Survival curves of adults of *L. botrana, G. molesta* and *C. pomonella* subjected to different feeding regimes.

Table S1. Statistical analysis for the size (length and width) of the apical segment of the labial palp. a) Model selection showing the Akaike information criterion (AIC). b) Summary of the selected models. c) Pairwise comparison among species and between males and females showing observed values and those estimated by the models. Different letters in the column to the right indicate significant differences among groups (Tukey´s test, P<0.05 after GLM).

a

Model (Length)	AIC	DF model	DF residual deviance	Residual deviance	LRT P-value
Null	655	0	59	180144	-
Main effects	529	3	56	20077	<0.001
Main effects and 2nd-order interaction	512	5	54	14194	<0.001

Model (Width)	AIC	DF model	DF residual deviance	Residual deviance	LRT P-value
Null	567	0	59	42073	-
Main effects	456	3	56	5920	<0.001
Main effects and 2nd-order interaction	437	5	54	4073	<0.001

b

Length	Df	Deviance	Resid.Df	Resid.Dev	F	Pr(>F)
NULL			59	180144		
sp	2	119116.00	57	61028	226.58	<0.001
sex	1	40951.00	56	20077	155.79	<0.001
sp:sex	2	5883.00	54	14194	11.19	<0.001

Width	Df	Deviance	Resid.Df	Resid.Dev	F	Pr(>F)
NULL			59	42073		
Sp	2	24946.50	57	17127	165.39	<0.001
sex	1	11206.70	56	5920	148.60	<0.001
sp:sex	2	1847.70	54	4073	12.25	<0.001

c

Length	Observed			Estimated			
	Mean ± SEM			Mean ± SEM			
C. pomonella	240.13	±	9.87	240.13	±	3.63	a
L. botrana	210.63	±	6.83	210.63	±	3.63	b
G. molesta	134.38	±	4.06	134.38	±	3.63	c
Female	221.17	±	2.96	221.17	±	10.65	a
Male	168.92	±	2.96	168.92	±	6.82	b

Width	Observed			Estimated			
	Mean ± SEM			Mean ± SEM			
C. pomonella	129.50	±	3.63	129.50	±	1.94	a
G. molesta	86.63	±	2.66	86.63	±	1.94	b
L. botrana	85.88	±	4.98	85.88	±	1.94	b
Female	114.33	±	4.23	114.33	±	1.59	a
Male	87.00	±	4.19	87.00	±	1.59	b

Table S2. Statistical analysis for the number of sensilla in the apical segment of the labial palp. a) Model selection showing the Akaike information criterion (AIC). Main effects: species and sex. b) Summary of the selected model. c) Pairwise comparison among species and between males and females showing observed values and those estimated by the models. Different letters in the column to the right indicate significant differences among groups (Tukey´s test, P<0.05 after GLM).

a

Model	AIC	DF model	DF residual deviance	Residual deviance	LRT P-value
Null	280	0	59	37	-
Main effects	268	3	56	19	<0.001
Main effects and 2nd-order interaction	270	5	54	17	0.345

b

	Df	Deviance	Resid. Df	Resid. Dev	Pr(>Chi)
NULL			59	37.09	
sp	2	9.57	57	27.52	0.008
sex	1	8.41	56	19.10	0.004

c

	Observed			Estimated			
	Mean ± SEM			Mean ± SEM			
Female	10.03	±	0.46	9.94	±	0.58	a
Male	7.80	±	0.30	7.73	±	0.51	b
C. pomonella	10.50	±	0.55	10.42	±	0.72	a
L. botrana	8.65	±	0.44	8.58	±	0.65	ab
G. molesta	7.60	±	0.40	7.54	±	0.61	b

Table S3. Statistical analysis for the effect of labial palp location on the number of sensilla chaetica in each moth species. a) Model selection showing the Akaike information criterion (AIC). Main effects: palp location. b) Summary of the selected models. c) Pairwise comparison among palp locations showing observed values and those estimated by the models. Different letters in the column to the right indicate significant differences among groups (Tukey´s test, P<0.05 after GLM). * The location code can be found in Supplementary Fig. S1.

a

Species	Model type	AIC	DF model	DF residual deviance	Residual deviance	LRT P-value*
L. botrana	Null	454	0	159	222	-
	Main effects	345	7	152	94	<0.001
G. molesta	Null	488	0	159	312	-
	Main effects	293	7	152	103	<0.001
C. pomonella	Null	542	0	159	301	-
	Main effects	362	7	152	107	<0.001

b

Species		Model Df	Deviance	Resid. Df	Resid. Dev	Pr(>Chi)
L. botrana	NULL			159	221.57	
	loc	7	123.66	152	97.91	<0.001
G. molesta	NULL			159	311.93	
	loc	7	208.53	152	103.40	<0.001
C. pomonella	NULL			159	301.09	
	loc	7	193.74	152	107.35	<0.001

c

		Observed			Estimated			
Species	Location*	Mean ± SEM			Mean ± SEM			
L. botrana	I	3.00	±	0.18	3.00	±	0.39	a
	II	1.75	±	0.20	1.75	±	0.30	ab
	IV	1.30	±	0.18	1.30	±	0.25	bc
	III	1.30	±	0.19	1.30	±	0.25	bc
	VIII	0.55	±	0.15	0.55	±	0.17	cd
	VI	0.50	±	0.14	0.50	±	0.16	cd
	V	0.15	±	0.08	0.15	±	0.09	d
	VII	0.10	±	0.07	0.10	±	0.07	d
G. molesta	I	3.80	±	0.24	3.80	±	0.44	a
	III	1.95	±	0.21	1.95	±	0.31	b
	V	0.75	±	0.14	0.75	±	0.19	c
	VII	0.45	±	0.20	0.45	±	0.15	c
	II	0.40	±	0.15	0.40	±	0.14	c
	VI	0.10	±	0.07	0.10	±	0.07	c
	IV	0.10	±	0.07	0.10	±	0.07	c
	VIII	0.05	±	0.05	0.05	±	0.05	c
C. pomonella	I	3.65	±	0.33	3.65	±	0.43	a
	II	2.85	±	0.26	2.85	±	0.38	ab
	III	1.65	±	0.22	1.65	±	0.29	bc
	V	1.20	±	0.21	1.20	±	0.24	c
	IV	0.70	±	0.18	0.70	±	0.19	cd
	VI	0.25	±	0.10	0.25	±	0.11	d
	VII	0.10	±	0.07	0.10	±	0.07	d
	VIII	0.10	±	0.07	0.10	±	0.07	d

Table S4. Number of moths and number of sensilla tested in the SSR experiment.

Species	sex	Stimulus	Appendage	N. moths	N. sensilla
L. botrana	Female	KCl	Palp	5	21
L. botrana	Female	KCl	Antenna	4	23
L. botrana	Female	NaCl	Palp	4	22
L. botrana	Female	NaCl	Antenna	4	24
L. botrana	Female	Fructose	Palp	4	22
L. botrana	Female	Fructose	Antenna	4	22
L. botrana	Female	Sucrose	Palp	4	20
L. botrana	Female	Sucrose	Antenna	4	22
L. botrana	Male	KCl	Palp	5	25
L. botrana	Male	KCl	Antenna	4	19
L. botrana	Male	NaCl	Palp	4	24
L. botrana	Male	NaCl	Antenna	4	21
L. botrana	Male	Fructose	Palp	5	25
L. botrana	Male	Fructose	Antenna	4	20
L. botrana	Male	Sucrose	Palp	4	19
L. botrana	Male	Sucrose	Antenna	5	28
G. molesta	Female	KCl	Palp	4	22
G. molesta	Female	KCl	Antenna	4	23
G. molesta	Female	NaCl	Palp	4	23
G. molesta	Female	NaCl	Antenna	4	19
G. molesta	Female	Fructose	Palp	4	21
G. molesta	Female	Fructose	Antenna	4	21
G. molesta	Female	Sucrose	Palp	4	20
G. molesta	Female	Sucrose	Antenna	4	22
G. molesta	Male	KCl	Palp	4	20
G. molesta	Male	KCl	Antenna	4	16
G. molesta	Male	NaCl	Palp	4	19
G. molesta	Male	NaCl	Antenna	6	34
G. molesta	Male	Fructose	Palp	4	23
G. molesta	Male	Fructose	Antenna	5	29
G. molesta	Male	Sucrose	Palp	4	19
G. molesta	Male	Sucrose	Antenna	5	24
C. pomonella	Female	KCl	Palp	5	27
C. pomonella	Female	KCl	Antenna	5	24
C. pomonella	Female	NaCl	Palp	5	18
C. pomonella	Female	NaCl	Antenna	4	22
C. pomonella	Female	Fructose	Palp	4	21
C. pomonella	Female	Fructose	Antenna	4	24
C. pomonella	Female	Sucrose	Palp	4	18
C. pomonella	Female	Sucrose	Antenna	4	18
C. pomonella	Male	KCl	Palp	5	28
C. pomonella	Male	KCl	Antenna	4	20
C. pomonella	Male	NaCl	Palp	5	25
C. pomonella	Male	NaCl	Antenna	4	19
C. pomonella	Male	Fructose	Palp	4	20
C. pomonella	Male	Fructose	Antenna	4	20
C. pomonella	Male	Sucrose	Palp	4	20
C. pomonella	Male	Sucrose	Antenna	4	21

Table S5. Comparison between each of the 3 sugars concentrations and the electrolyte control for the number of spikes of sensilla chaetica on the antenna and labial palps. a) Model selection. Main paramet[illegible] are: species, sex and appendage. b) GLM model summary. c) P-values of the Dunnett´s test after GLM ar[illegible] shown. Comparison between each test concentrations (10^1, 10^2 and 10^3 mM) of sugars and the solv[illegible] control in each.

a

Model	DF model	DF Resid. Dev.	Resid. Dev.	P-Val[illegible]
Null		2075.00	13801.15	
Main effects	8	2067.00	10847.75	<[illegible]
Main effects and 2nd-order interaction	32	2043.00	9663.03	<[illegible]
Main effects and 2nd- and 3rd-order interaction	66	2009.00	9144.20	<[illegible]
Main effects and 2nd-, 3rd- and 4rt-order interaction	89	1986.00	8921.24	[illegible]
Main effects and 2nd-, 3rd- , 4rt- and 5th order interaction	95	1980.00	8829.49	[illegible]

b

	DF	Deviance	Resid. Df	Resid. Dev	F	Pr(>F)
NULL			2075	13801.20		
sp	2	167.87	2073	13633.30	15.81	<0.01
sex	1	198.92	2072	13434.40	37.47	<0.01
app	1	1798.54	2071	11635.80	338.74	<0.01
stim	1	452.33	2070	11183.50	85.19	<0.01
conc	3	335.74	2067	10847.70	21.08	<0.01
sp:sex	2	237.07	2065	10610.70	22.32	<0.01
sp:app	2	186.93	2063	10423.80	17.60	<0.01
sex:app	1	61.48	2062	10362.30	11.58	<0.01
sp:stim	2	131.27	2060	10231.00	12.36	<0.01
sex:stim	1	0.13	2059	10230.90	0.02	0.87
app:stim	1	167.69	2058	10063.20	31.58	<0.01
sp:conc	6	50.73	2052	10012.50	1.59	0.15
sex:conc	3	1.36	2049	10011.10	0.09	0.97
app:conc	3	296.01	2046	9715.10	18.58	0.00
stim:conc	3	52.05	2043	9663.00	3.27	0.02
sp:sex:app	2	73.04	2041	9590.00	6.88	<0.01
sp:sex:stim	2	94.83	2039	9495.20	8.93	<0.01
sp:app:stim	2	108.52	2037	9386.60	10.22	<0.01
sex:app:stim	1	0.49	2036	9386.20	0.09	0.76
sp:sex:conc	6	53.81	2030	9332.30	1.69	0.12
sp:app:conc	6	84.80	2024	9247.50	2.66	0.01
sex:app:conc	3	16.88	2021	9230.70	1.06	0.36
sp:stim:conc	6	8.27	2015	9222.40	0.26	0.96
sex:stim:conc	3	37.21	2012	9185.20	2.34	0.07
app:stim:conc	3	40.97	2009	9144.20	2.57	0.05
sp:sex:app:stim	2	56.89	2007	9087.30	5.36	0.00
sp:sex:app:conc	6	17.52	2001	9069.80	0.55	0.77
sp:sex:stim:conc	6	53.07	1995	9016.70	1.67	0.13
sp:app:stim:conc	6	62.59	1989	8954.10	1.96	0.07
sex:app:stim:conc	3	32.90	1986	8921.20	2.07	0.10
sp:sex:app:stim:conc	6	91.74	1980	8829.50	2.88	0.01

c

Appendage	Stimulus	Species	Sex	10^1 mM	10^2 mM	10^3 mM
Antenna	Fructose	*L. botrana*	Female	0.552	0.980	0.088
			Male	0.474	0.567	0.889
		G. molesta	Female	0.998	0.865	0.337
			Male	0.421	0.113	0.118
		C. pomonella	Female	0.999	0.393	0.393
			Male	0.769	0.340	0.036
	Sucrose	*L. botrana*	Female	0.453	0.998	0.673
			Male	0.998	0.998	0.487
		G. molesta	Female	0.930	0.851	0.564
			Male	0.840	0.787	0.634
		C. pomonella	Female	0.995	0.144	0.925
			Male	0.421	0.474	0.559
Palp	Fructose	*L. botrana*	Female	0.245	0.028	0.026
			Male	0.342	0.845	0.096
		G. molesta	Female	0.990	0.554	0.737
			Male	0.805	0.991	0.999
		C. pomonella	Female	0.866	0.765	0.631
			Male	0.729	0.099	0.180
	Sucrose	*L. botrana*	Female	<0.001	<0.001	<0.001
			Male	0.222	0.134	0.203
		G. molesta	Female	0.474	0.016	0.003
			Male	0.013	<0.001	0.032
		C. pomonella	Female	0.328	0.003	0.002
			Male	0.660	0.197	0.086

Table S6. Model selection and pairwise comparisons for the number of spikes produced by sensilla chaetica on the labial palps and antennae. The main effects were sex, species (*C. pomonella*, *G. molesta* and *L. botrana*), appendage (antenna or palp), stimulus (fructose, sucrose, NaCl and KCl, or sugars and salts combined), and concentration (10^1, 10^2 and 10^3 mM). a). Models were compared with ANOVA (p-values of the likelihood ratio test, LRT). b) Pairwise comparison among concentrations for salts and sugars. c) Pairwise comparison among salts and sugars for palp and antenna. d) Pairwise comparison among salts and sugars for each species. e) Pairwise comparison between sexes for each species. f) Pairwise comparison between appendages for salts and sugars.

a

Model	DF model	DF Res. Dev.	Res. dev.	LRT P-value
Null	0	3170	23956	-
Main effects	9	3161	19497	<0.001
Main effects and 2nd-order interaction	40	3130	16776	<0.001
Main effects and 2nd- and 3rd-order interaction	91	3079	15539	<0.001
Main effects and 2nd-, 3rd- and 4rt-order interaction	131	3039	15079	0.001
Main effects and 2nd-, 3rd- , 4rt- and 5th order interaction	143	3027	14994	0.314

b

		Observed			Estimated			
mM	Stimulus	Mean	±	SEM	Mean	±	SEM	
	10^1	1.86	±	0.12	1.34	±	0.15	b
Salts	10^2	0.97	±	0.12	0.26	±	0.12	c
	10^3	5.34	±	0.41	3.96	±	0.26	a
	10^1	2.89	±	0.22	2.13	±	0.19	a
Sugars	10^2	3.67	±	0.28	2.40	±	0.22	a
	10^3	3.28	±	0.27	1.82	±	0.21	a

c

		Observed			Estimated			
Appendage	Stimulus	Mean	±	SEM	Mean	±	SEM	
Palp	Sugars	5.33	±	0.27	4.34	±	0.22	a
	Salts	3.60	±	0.27	1.97	±	0.17	b
Antenna	Sugars	1.40	±	0.11	1.02	±	0.10	c
	Salts	1.81	±	0.14	0.63	±	0.19	c

d

		Observed			Estimated			
Stimulus	Species	Mean	±	SEM	Mean	±	SEM	
	C. pomonella	4.15	±	0.34	1.52	±	0.33	a
Salts	*L. botrana*	2.09	±	0.23	0.99	±	0.22	a
	G. molesta	1.89	±	0.20	0.91	±	0.19	a
	C. pomonella	4.02	±	0.27	2.93	±	0.24	a
Sugars	*L. botrana*	3.23	±	0.23	2.30	±	0.21	a
	G. molesta	2.66	±	0.27	1.38	±	0.17	b

e

		Observed			Estimated			
Species	sex	Mean	±	SEM	Mean	±	SEM	
L. botrana	Female	3.39	±	0.22	1.98	±	0.28	a
	Male	1.95	±	0.24	1.15	±	0.19	b
G. molesta	Female	2.64	±	0.24	1.60	±	0.18	a
	Male	1.94	±	0.24	0.78	±	0.16	b
C. pomonella	Female	4.07	±	0.31	2.17	±	0.35	a
	Male	4.11	±	0.31	2.06	±	0.30	a

f

			Observed			Estimated			
Stimulus	Appendage	mM	Mean	±	SEM	Mean	±	SEM	
		10^1	1.69	±	0.16	1.27	±	0.21	b
	Antenna	10^2	0.43	±	0.12	0.07	±	0.06	c
		10^3	3.33	±	0.37	2.76	±	0.30	a
Salts		10^1	2.03	±	0.18	1.41	±	0.22	b
	Palp	10^2	1.50	±	0.21	0.95	±	0.18	b
		10^3	7.28	±	0.71	5.68	±	0.41	a
		10^1	1.83	±	0.19	1.44	±	0.21	a
	Antenna	10^2	1.40	±	0.18	1.12	±	0.18	ab
		10^3	0.97	±	0.21	0.66	±	0.14	b
Sugars		10^1	4.05	±	0.39	3.16	±	0.34	b
	Palp	10^2	6.15	±	0.51	5.12	±	0.41	a
		10^3	5.79	±	0.48	5.07	±	0.39	a
